# Thymic Involution and Altered Naive CD4 T Cell Homeostasis in Neuromyelitis Optica Spectrum Disorder

**DOI:** 10.3389/fimmu.2021.645277

**Published:** 2021-07-16

**Authors:** Haoxiao Chang, Hengri Cong, Huabing Wang, Li Du, De-Cai Tian, Yuetao Ma, Yun Xu, Yupeng Wang, Linlin Yin, Xinghu Zhang

**Affiliations:** ^1^ Department of Neurology, Beijing Tiantan Hospital, Capital Medical University, Beijing, China; ^2^ China National Clinical Research Center for Neurological Diseases, Beijing, China; ^3^ Advanced Innovation Center for Human Brain Protection, Beijing, China

**Keywords:** neuromyelitis optica spectrum disorder, thymic involution, CT, naive T cells, flow cytometry

## Abstract

Circulating T helper cells with a type 17-polarized phenotype (TH17) and expansion of aquaporin-4 (AQP4)-specific T cells are frequently observed in patients with neuromyelitis optica spectrum disorder (NMOSD). However, naive T cell populations, which give rise to T helper cells, and the primary site of T cell maturation, namely the thymus, have not been studied in these patients. Here, we report the alterations of naive CD4 T cell homeostasis and the changes in thymic characteristics in NMOSD patients. Flow cytometry was performed to investigate the naive CD4^+^ T cell subpopulations in 44 NMOSD patients and 21 healthy controls (HC). On immunological evaluation, NMOSD patients exhibited increased counts of CD31^+thymic^ naive CD4^+^ T cells and CD31^-cental^ naive CD4^+^ T cells along with significantly higher fraction and absolute counts of peripheral blood CD45RA^+^ CD62L^+^ naive CD4^+^ T cells. Chest computed tomography (CT) images of 60 NMOSD patients and 65 HCs were retrospectively reviewed to characterize the thymus in NMOSD. Thymus gland of NMOSD patients exhibited unique morphological characteristics with respect to size, shape, and density. NMOSD patients showed exacerbated age-dependent thymus involution than HC, which showed a significant association with disease duration. These findings broaden our understanding of the immunological mechanisms that drive severe disease in NMOSD.

## Introduction

Neuromyelitis optica spectrum disorder (NMOSD) is a severe disabling autoimmune disease of the central nervous system (CNS) associated with anti-aquaporin-4 (AQP4)-autoantibody. T helper subsets, which affect the differentiation of B cells into antibody-producing cells, have been implicated in the pathogenesis of this autoimmune disorder (1). Changes in the circulating T-cell profile indicate a key role of T cells in NMOSD (2, 3). Thymus is the primary lymphoid organ, which regulates the development, selection, and maturation of T cells (4). However, alterations in the thymic characteristics or thymic function, if any, in NMOSD have not been elucidated.

Naive T cells are generated in the thymus and recirculate among lymphoid organs. Exposure of naïve T cells to foreign antigens induces their activation and differentiation into effector and memory T cells (5, 6). Preservation of the peripheral naive T cell pool in humans requires both thymic output and homeostatic proliferation (7–9). Interleukin-6 (IL-6) can promote the differentiation of naive T cells into proinflammatory type 17 helper T cells, which, along with interleukin-6, promote the differentiation of B cells into AQP4-IgG–producing plasmablasts (10–12). This phenomenon consistently aggravates the disease severity in patients with NMOSD. However, the naive T cell homeostasis in NMOSD patients is not well characterized.

Naive T cells are regarded as a fairly homogeneous and quiescent cell population, differing only with respect to T-cell receptor (TCR) specificity; the size of the naive T cell population depends on age and thymic output (13). Phenotypically, human naive CD4 ^+^ T cells express surface markers, such as CCR7, CD45RA, CD62L, CD27, and CD28 (14, 15). Previous studies have shown that surface CD31 expression distinguishes two subpopulations of CD45RA^+^ CD62L^+^ naive CD4^+^ T cells with distinct T-cell receptor excision circle (TREC) content in the peripheral blood of humans (13, 14, 16). CD31^+ thymic^ naive CD4^+^ T cells were found to be enriched in TRECs, comprising of recent thymic emigrants, whereas CD31^- central^ naive CD4^+^ T cells displayed a rather low TREC content, characterized by striking TCR repertoire restrictions, seemingly generated by homeostatic proliferation of naive CD4^+^ T cells (13). The fraction and absolute count of CD31^+ thymic^ naive CD4^+^ T cells show a negative correlation with age and the age-related decline in thymic function, which can be regarded as direct marker of thymic output (17). In contrast, the absolute number of CD31^-central^ naive CD4^+^ T cells tends to remain stable over time, implying a peripheral regulation independent of thymic activity (18).

Thymic homing of activated CD4^+^ T cells has been shown to induce the degeneration of thymus gland (19); therefore, the thymic characteristics and thymic function in NMOSD were unexpected observations on account of the CD4^+^ TH17 cells polarization. This complexity provides interesting insights into the pathogenesis of NMOSD.

In this study, we retrospectively reviewed the chest computed tomography (CT) images to describe the characteristics of thymus in patients with NMOSD and measured the thymic density as a representative marker of thymic function. In addition, we performed flow cytometric analysis of naive T cells to investigate the homeostasis alteration and thymic output.

## Methods

### Patient Selection

#### Flow Cytometry

Forty-four patients with NMOSD, who were diagnosed according to 2015 International Panel for Neuromyelitis Optica Diagnostic criteria at the Neurology department of the Beijing Tiantan Hospital between August 2019 and January 2020, and 21 healthy controls (HC) from the Health Management Center underwent flow cytometry to investigate the naive CD4^+^ T cell subpopulations. All patients with NMOSD were in the acute phase, and the samples were collected prior to treatment. None of the NMOSD patients had any other autoimmune disease. Eligible HC had no history of autoimmune diseases, thymoma, or other underlying diseases. Women who were pregnant or breastfeeding within the last 6 months were ineligible for this study. The demographic and clinical characteristics of all participants are summarized in **Table 1**.

**Table 1:** Clinical and demographic characteristics of participants who underwent flow cytometric analysis.

Characteristic	NMOSD	HC	*P* Value
Number	44	21	
Age^a^, mean (SD), y	42.8 (15.8)	39.1 (14.7)	0.42
Sex^b^, No. (%)			
Male	10 (22.7%)	1 (4.8%)	0.985
Female	34 (77.3%)	20 (95.2%)	
Disease duration, mean (range), m	31.9 (0.5–205.0)	**–**	
Relapse times, median (interquartile range)	3.8 (3, 5)	**–**	
EDSS at FCM analysis, median (interquartile range)	4.2 (3.0, 6.0)	**–**	
Antibody status (Cell based assay)			
AQP4-IgG, No. (%)	31 (70.5%)	–	

a Mann-Whitney U test.

b Differences of sex and thymus shapes between groups were assessed using Chi-squared test. y, year; m, month; FCM, flow cytometry; NMOSD, Neuromyelitis optica spectrum disorder; HC, healthy controls; EDSS, Expanded Disability Status Scale.

#### Chest Computed Tomography

We retrospectively reviewed chest CT images of 60 patients with NMOSD from the Neurology Department, Beijing Tiantan Hospital, from May 2018 to December 2019, and 65 age-, sex-matched healthy adults from the Health Management Center, Beijing Tiantan Hospital as HC. Eligible HC with no history of autoimmune diseases, thymoma, or other underlying diseases were enrolled for chest CT examination. Women who were pregnant or breastfeeding within the last 6 months were ineligible for this study. The demographic and thymus characteristics of all participants are summarized in **Table 3**.

### Image Acquisition and Analysis

All imaging was conducted on a 256 slice Discovery CT750 HD CT Scanner (GE Healthcare, Waukesha, WI). Chest CT scans with axial technique 5-mm slice thickness were evaluated on a Picture Archiving and Communication System (PACS) workstation (Neusoft Co., China). All images were reviewed on a PACS, using a mediastinal window setting (level, 50 HU; width, 350 HU).

### Flow Cytometric Analysis of Naive CD4 T Cells Subpopulations

Whole blood samples (100 μl) were stained in TruCount™ tubes with anti-CD45RA, CD3, CD4, CD31, and CD62L antibodies (Biolegend) after red blood cell lysis. The CytExpert software (Beckman Coulter) was used for analysis.

### Serum IL-6 Analysis in the Population Subjected to Flow Cytometry

After flow cytometric analysis, blood samples were centrifuged to collect the serum.

Serum IL-6 was measured using a human IL-6 Quantikine ELISA Kit according to the manufacturer’s instructions (R&D systems D6050).

### Statistical Analysis

Data are presented as mean (standard deviation), median (interquartile range), or frequency (%), as appropriate. Independent-samples *t* test or Mann–Whitney *U* test was used to analyze differences between NMOSD and HC, with respect to scalar and nonparametric parameters. The chi-squared test was used to examine the differences of sex and thymus shapes between groups. Linear regressions were performed to examine the relationships of the frequency and absolute counts of naive CD4^+^ T-cell subpopulations with age and sex. Statistical analysis of thymic predominant sides, volume, density, and thymic scores was performed after disaggregating patients by age, sex, and body mass index (BMI). Mediation effects were analyzed by SPSS Statistics 26.0 plug-in PROCESS V3.4. *P* values less than 0.05 were considered indicative of statistical significance.

## Results

### NMOSD Patients Displayed Higher Fraction and Count of Naive CD4 T Cells

**Figure 1:**
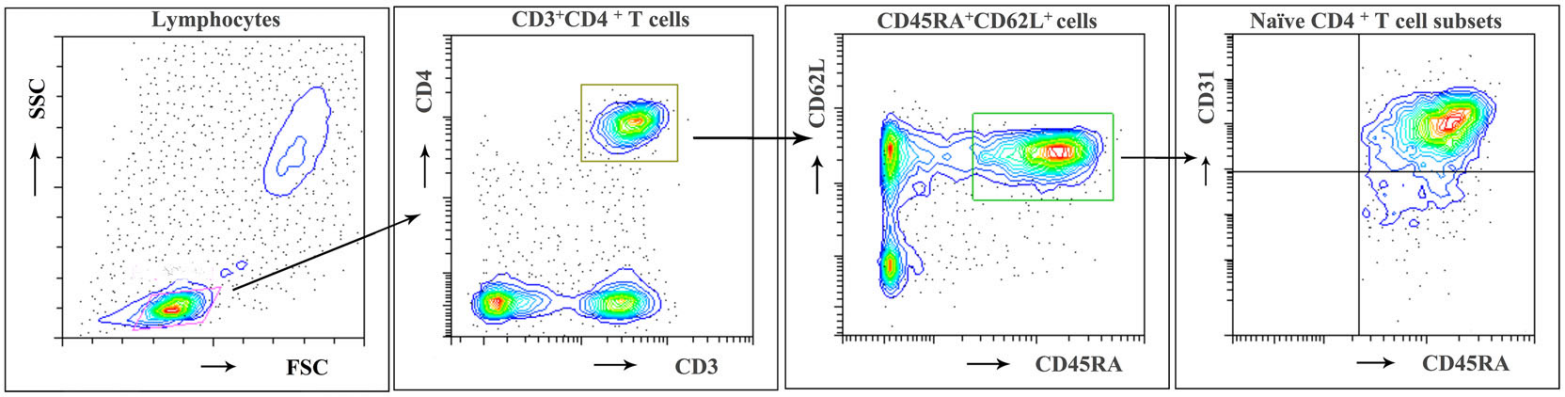
Flow cytometric identification of naïve CD4 + T cell subpopulations. A representative analysis of CD31^+ thymic^ naive and CD31^- central^ naive CD4 + T cells as described in *Materials and Methods* is depicted. First lymphocytes were identified according to FSC/SSC characteristics. Then CD3^+^ CD4^+^ cells were gated and subdivided according to CD62L and CD45RA expression. Among CD62L^+^ and CD45RA^+^ naive CD4^+^ T cells, the frequency of CD31^+ thymic^ naive and CD31^−central^ naive CD4^+^ T cells was determined. Representative results of a 21-year-old woman from the HC group.

**Figure 2:**
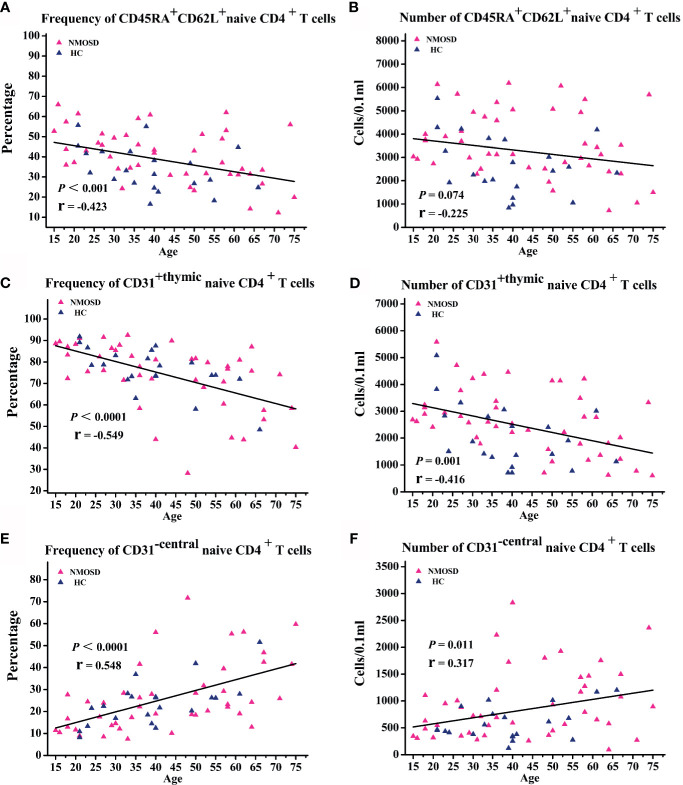
Correlation analysis of naive T cell subpopulations with age. **(A–F)** Scatter plots showing the correlation of naive T cell subpopulations with age. NMOSD, Neuromyelitis optica spectrum disorder; HC, healthy controls.

Flow cytometry was used to investigate the naive CD4^+^ T cell subpopulations in 44 NMOSD patients (mean age, 42.8 years; range, 15–75) and 21 HC. A representative analysis is shown in **Figure 1**. Compared with HC, NMOSD patients displayed significantly higher fraction of peripheral blood CD45RA^+^ CD62L^+^ naive CD4^+^ T cells (95% CI, −13.811 to −1.487; *P* = 0.016; **Table 2**) which showed an age-dependent decrease (*P* < 0.001; r = −0.423; **Figure 2A**). The absolute numbers of CD45RA^+^ CD62L^+^ naive CD4^+^ T cells in NMOSD patients was also significantly higher than that in HC (95% CI, −1735.694 to −284.597; *P* = 0.007; **Table 2**) and showed a declining trend with increase in age, although not statistically significant (*P* = 0.074; r = −0.225; **Figure 2B**). No significant difference was observed in naive CD4 T cells subpopulations between anti-AQP4-positive and anti-AQP4-negative patients.

**Table 2:** Linear regressions analysis between multiple variables and naïve T cells.

	B	SE	95% CI	*P* value
Frequency of CD45RA^+^ CD62L^+^ naive CD4^+^ T cells				
NMOSD *vs* HC	-7.649	3.082	-13.811 – -1.487	0.016
Age	-0.353	0.089	-0.531 – -0.176	<0.001
Sex	1.068	3.92	-6.770 – 8.905	0.786
Number of CD45RA^+^ CD62L^+^ naive CD4^+^ T cells				
NMOSD *vs* HC	-1010.145	362.843	-1735.694 – -284.597	0.007
Age	-21.803	10.467	-42.733 – -0.873	0.041
Sex	-137.891	461.509	-1060.735 – 784.952	0.766
Frequency of CD31^+thymic^ naive CD4^+^ T cells				
NMOSD *vs* HC	0.537	3.256	-5.973 – 7.048	0.87
Age	-0.48	0.094	-0.668 – -0.292	<0.001
Sex	-1.501	4.141	-9.781 – 6.780	0.718
Number of CD31^+thymic^ naive CD4^+^ T cells				
NMOSD *vs* HC	-703.577	291.031	-1285.531 – -121.624	0.019
Age	-32.627	8.396	-49.415 – -15.839	<0.001
Sex	-68.831	370.17	-809.032 – 671.370	0.853
Frequency of CD31^-central^ naïve CD4^+^ T cells				
NMOSD *vs* HC	-0.485	3.257	-6.998 – 6.028	0.882
Age	0.479	0.094	0.291 – 0.667	<0.001
Sex	1.503	4.143	-6.781 – 9.787	0.718
Number of CD31^-cental^ naive CD4^+^ T cells				
NMOSD *vs* HC	-306.542	145.937	-598.362 – -14.722	0.04
Age	10.823	4.21	2.405 – 19.241	0.013
Sex	-69.028	185.621	-440.201 – 302.145	0.711

NMOSD, Neuromyelitis optica spectrum disorder; HC, healthy controls; B, regression coefficient; SE, standard error; CI, confidence interval.

### NMOSD Thymic Output Naive CD4 T Cells Homeostasis Analysis

CD31^+ thymic^ naive CD4^+^ T cells is a subpopulation of CD45RA ^+^ CD62L ^+^ naive CD4^+^ T cells and are regarded as direct marker of thymic output function (13, 20).

Because the fraction and absolute count of CD31^+ thymic^ naïve CD4^+^ T cells declines with increase in age (17), linear regressions were performed to examine the relationship of the frequency and absolute count of CD31^+ thymic^ naïve CD4^+^ T cells with age and sex. Correlation analysis showed an age-dependent decrease in the frequency of CD31^+ thymic^ naive CD4^+^ T cells (*P* < 0.0001; r = −0.549; **Figure 2C**) and absolute numbers of CD31^+ thymic^ naive CD4^+^ T cells (*P* = 0.001; r = −0.416; **Figure 2D**). NMOSD patients show significantly higher absolute numbers of CD31^+ thymic^ naïve CD4^+^ T cells (95% CI, -1285.531 – -121.624; *P* = 0.019; **Table 2**) than HC, whereas no changes were observed in the frequency of CD31^+ thymic^ naive CD4^+^ T cells. The frequency and absolute numbers of CD31^−central^ naive CD4^+^ T cells showed a significant positive correlation with age (*P* < 0.0001, r = 0.548; *P* = 0.011, r = 0.317; **Figures 2E, F** **)**. In addition, NMOSD patients displayed significantly higher absolute numbers of CD31^−central^ naive CD4^+^ T cells than HC (95% CI, −598.362 to −14.722; *P* = 0.04; **Table 2**).

We conclude that despite the absolute numbers of CD31^+ thymic^ naive CD4^+^ T cells and absolute numbers of CD31^-central^ naive CD4^+^ T cells showing opposite age-related trends, the absolute numbers of CD31^+ thymic^ naive CD4^+^ T cells and CD31^-central^ naive CD4^+^ T cells in patients seemed to increased proportionally, which may explain the lack of observed difference in the proportions. It is hard to draw conclusions about the NMOSD thymic output naive CD4 T cells changes, because only the absolute numbers were altered. In the absence of other parameters of thymic function, it may be controversial to assess thymus activity by thymic output naïve T cells alone.

### NMOSD Displayed Higher Level IL-6 and Significantly Correlate With Naive T Cells

**Figure 3:**
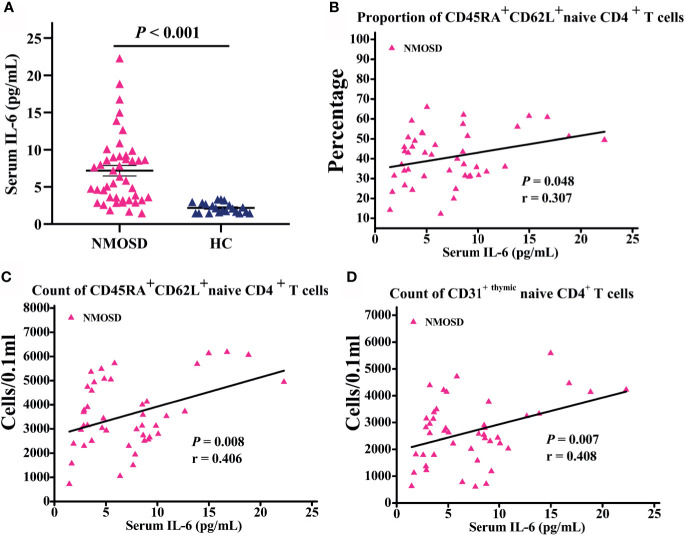
Serum IL-6 level in participants subjected to flow cytometric analysis and correlation analysis of naive T cells with IL-6 level. **(A)** IL-6 levels in NMOSD were significantly higher than HC; **(B–D)** Scatter plots showing the correlation of naive T cells with IL-6 level; r = partial correlation coefficient after for age and gender. NMOSD, neuromyelitis optica spectrum disorder; HC, healthy controls.

Serum samples of 44 patients and 21 HC were collected to detect the level of IL-6. Notably, IL-6 levels in the serum of NMOSD patients were significantly higher than those in HC (*P* < 0.001; **Figure 3A**). Moreover, partial correlation analysis after controlling for age and sex revealed a marked positive association between CD45RA^+^ CD62L^+^ naive CD4^+^ T cells proportion and IL-6 level (*P* = 0.048; r = 0.307; **Figure 3B**), and the count of CD45RA^+^ CD62L^+^ naive CD4^+^ T cells increases with IL-6 level significantly (*P* = 0.008; r = 0.406; **Figure 3C**). The subpopulation of CD45RA ^+^ CD62L ^+^ naive CD4^+^ T cells—CD31^+ thymic^ naive CD4^+^ T cells count increases with IL-6 significantly (*P* = 0.007; r = 0.408; **Figure 3D**). We observed no significant correlation of CD31^−central^ naive CD4^+^ T cells with IL-6 level (data not shown).

### Measurement of Thymic Characteristics

**Figure 4:**
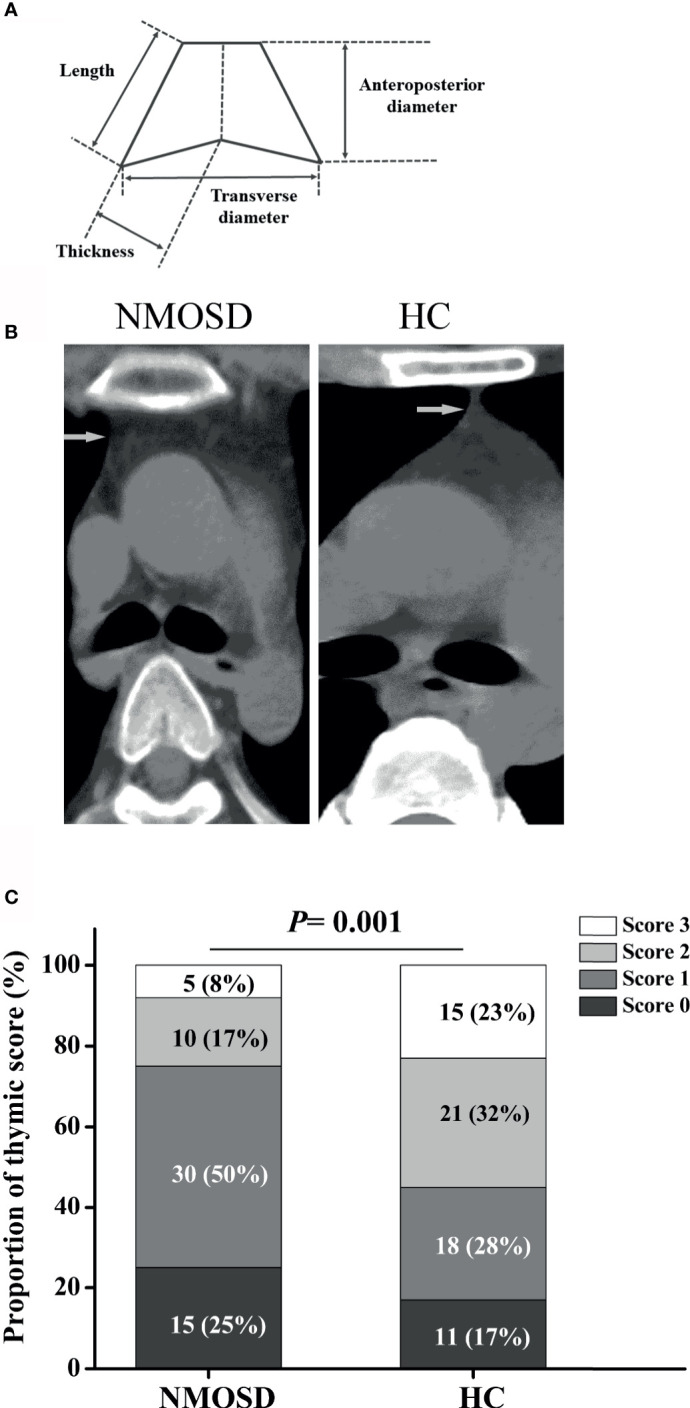
Schematic illustration of thymus measurements and the proportion of thymic scoring. **(A)** Schematic illustration of thymus measurement; **(B)** Representative axial CT images of thymus in age- and sex-matched NMOSD and HC groups (two 32-year-old women). **(C)** The proportion of thymic scoring, Score 0, complete fatty replacement and no identifiable soft tissue density in the thymic bed; Score 1, predominantly fatty thymus; Score 2, approximately one-half fatty and one half soft-tissue attenuated thymus; Score 3, predominantly soft-tissue attenuated thymus. NMOSD, Neuromyelitis optica spectrum disorder; HC, healthy controls.

The thymus gland has a unique morphology with a bilobed configuration, which can exhibit a pyramidal, triangular, arrowhead, or trapezoid shape on imaging. Therefore, morphological assessment of thymus requires dedicated methods specific for thymus (21). Parameters of thymus were measured as previously described (**Figure 4A**) (22, 23). Morphological evaluation was performed by an experienced technician who was blinded to the study using a thymic scoring system with a four-point scale (0-3) according to the proportion of fatty and soft tissues (24). CT density was measured by setting an oval region of interest covering the maximum area of the thymus gland with soft tissue density, excluding the surrounding mediastinal fatty tissue (22, 24). Predominant sides of thymus, density, and volume were analyzed blindly by two experienced radiologists. Linear and ordinal regressions were performed to examine the relationships of thymic predominant sides, volume, density, and thymic scores with age, sex, body mass index (BMI), and disease duration. For partial correlation coefficient, partial correlation analysis was performed after controlling for age or BMI.

### Predominant Size and Shape of the Thymus Differs in Healthy *vs.* NMOSD Patients and Is Impacted by BMI

Compared with HC, the trapezoid shape of the thymus gland was significantly more frequently observed in NMOSD patients (50% *vs* 32.3%; *P* = 0.044; **Table 3**). Representative axial CT images of the thymus with a different shape in NMOSD and HC were displayed in **Figure 4B**. The thymus of NMOSD patients exhibited distinct morphological changes characterized by significant increase in the left length, left thickness, right thickness, and transverse diameter of thymus (**Table 4**). However, there were no significant between-group differences with respect to right length and anteroposterior diameter (**Table 4**).

**Table 3:** Clinical, demographic, and thymus characteristics of participants evaluated by CT.

Characteristic	NMOSD (n=60)	HC (n=65)	*P* value
Age^a^, mean (SD), y	45.5 (15.8)	42.8 (14.7)	0.326
Sex^b^, No. (%)			
Male	11 (18.3%)	12 (18.5%)	0.985
Female	49 (81.7%)	53 (81.5%)	
BMI^c^, mean (SD)	23.7 (3.2)	23.6 (3.7)	0.875
Disease duration, mean (range), m	39.5 (0.5–194.0)	**–**	
Relapses, median (interquartile range)	3 (1, 4)	**–**	
EDSS at CT scan, median (interquartile range)	4.0 (2.0, 5.5)	**–**	
Relapse prevention treatment			
No relapse treatment	20		
Immunosuppressants	24		
B cell exhaustion	16		
Antibody status (Cell based assay)			
AQP4-IgG, seropositive, n (%)	44 (73.3%)	**–**	
Predominant sides of thymus^b^			
Trapezoid, n (%)	30 (50.0%)	21 (32.3%)	0.044
Other shapes, n (%)	30 (50.0%)	44 (67.7%)	
Left length, mean (range), mm	25.6 (11.6–44.2)	22.9 (11.0–38.7)	
Left thickness, mean (range), mm	11.9 (3.7–23.8)	8.4 (2.6–20.5)	
Right length, mean (range), mm	24.2 (7.5–48.1)	23.1 (10.7–40.8)	
Right thickness, mean (range), mm	12.1 (3.7–24.7)	8.9 (2.9–21.4)	
Transverse diameter, mean (range), mm	34.4 (10.4–67.2)	23.9 (7.3–52.7)	
Anteroposterior diameter, mean (range), mm	20.9 (7.8–42.2)	20.0 (8.9–33.9)	
Thymus density, mean (range), HU	-81.3 (-126–16)	-61.4 (-121–11)	

a Mann-Whitney U test.

b Differences of sex and thymus shapes between groups were assessed using Chi-squared test.

c Independent-samples t test. y, year; m, month; NMOSD, Neuromyelitis optica spectrum disorder; HC, healthy controls; BMI, body mass index; EDSS, Expanded Disability Status Scale.

**Table 4:** Linear regressions analysis between multiple variables and thymus parameter.

	B	SE	95% CI	*P* value
Predominant sides of thymus				
Left length				
NMOSD *vs* HC	-2.743	1.059	-4.839 – -0.646	0.011
Age	-0.022	0.037	-0.095 – -0.050	0.542
Sex	1.923	1.447	-0.943 – 4.789	0.186
BMI	1.117	0.165	0.789 – 1.444	<0.0001
Left thickness				
NMOSD *vs* HC	-3.476	0.598	-4.661 – -2.291	<0.0001
Age	0.02	0.021	-0.021 – 0.061	0.334
Sex	0.246	0.818	-1.374 – -1.866	0.764
BMI	0.473	0.093	0.288 – 0.658	<0.0001
Right length				
NMOSD *vs* HC	-1.227	1.192	-3.587 – 1.133	0.305
Age	-0.069	0.041	-0.151 – 0.012	0.096
Sex	1.867	1.63	-1.360 – 5.094	0.254
BMI	1.035	0.186	0.667 – 1.404	<0.0001
Right thickness				
NMOSD *vs* HC	-3.037	0.612	-4.248 – -1.826	<0.0001
Age	0.008	0.021	-0.034 – 0.050	0.714
Sex	0.534	0.836	-1.121 – 2.189	0.524
BMI	0.542	0.095	0.353 – 0.731	<0.0001
Transverse diameter				
NMOSD *vs* HC	-10.173	1.722	-13.582 – -6.763	<0.0001
Age	0.052	0.059	-0.065 – 0.170	0.379
Sex	-0.972	2.354	-5.633 – 3.688	0.68
BMI	1.528	0.269	0.996 – 2.060	<0.0001
Anteroposterior diameter				
NMOSD *vs* HC	-0.937	0.923	-2.763 – 0.890	0.312
Age	-0.055	0.032	-0.118 – 0.008	0.086
Sex	2.115	1.261	-0.382 – 4.612	0.096
BMI	0.941	0.144	0.656 – 1.226	<0.0001
Thymus density				
NMOSD *vs* HC	15.601	4.592	6.509 – 24.693	0.001
Age	-1.463	0.159	-1.777 – -1.149	<0.0001
Sex	6.521	6.277	-5.907 – 18.949	0.301
BMI	-2.69	0.717	-4.109 – -1.271	<0.001

NMOSD, Neuromyelitis optica spectrum disorder; HC, healthy controls; BMI, body mass index; B, regression coefficient; SE, standard error; CI, confidence interval.

**Figure 5:**
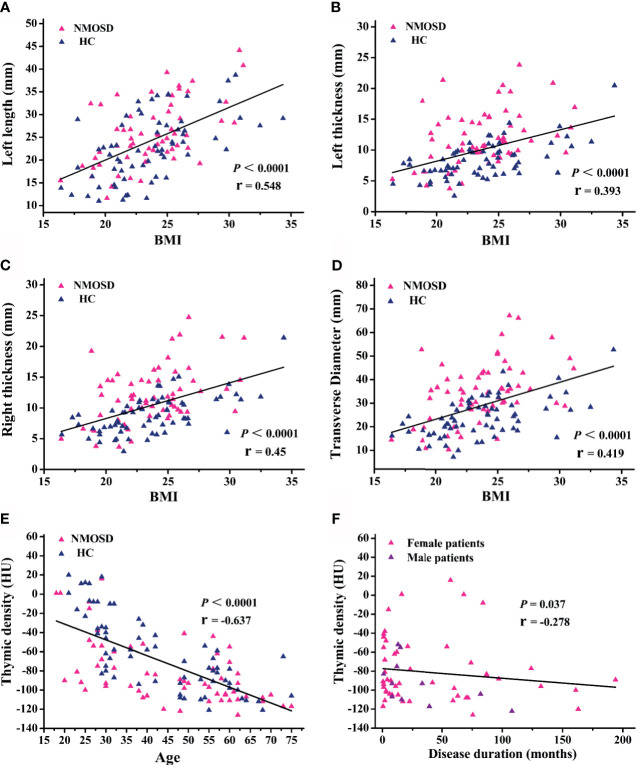
Regression and partial correlation analysis of thymic predominant sides, density, and disease duration. **(A–D)** Scatter plots showing the correlation of thymic predominant sides with body mass index (BMI); r = partial correlation coefficient after controlling for age. **(E)** Scatter plots showing correlation of thymic density with age; r = partial correlation coefficient after controlling for BMI. **(F)** Association between thymic density and disease duration; r = partial correlation coefficient after for age and BMI. NMOSD, neuromyelitis optica spectrum disorder; HC, healthy controls.

**Figure 6:**
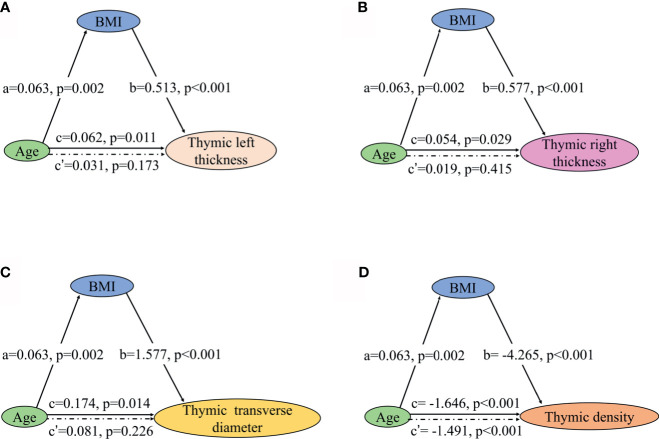
Mediation effects of changes in BMI on the association of increase in thymic predominant sides with age and decline in thymic density with age. **(A–C)** Mediation analysis was performed to examine the potential indirect relationship between Age (X) and thymic predominant sides (Y) via the BMI (M). **(D)** Mediation analysis the potential indirect relationship between Age (X) and thymic density (Y) *via* the BMI (M). Path a and path b indicate the association between X and M, and M and Y, respectively. Path c represents the total effect of X on Y, and path c’ shows the direct effect of X on Y after controlling for mediating factor. X, predictor variable; Y, outcome variable; M, mediator; BMI, body mass index.

Partial correlation analysis showed a positive correlation between thymic predominant sides and BMI (**Figures 5A–D**). Given the significant positive correlation between BMI and age (r = 0.278, *P* = 0.002), partial correlation coefficient of these thymic predominant sides was calculated according to age and BMI. Compared with age, BMI showed a direct association with thymic predominant sides. In the mediation analysis, BMI was found to fully mediate the association between increase in thymic predominant sides with age (**Figures 6A–C**) and to partially mediate the association between density decline with age (**Figure 6D**). No significant correlation was observed between sex and thymic predominant sides.

### NMOSD Patients Exhibited Higher Thymic Involution as Measured by Fatty Replacement of Thymic Tissue and Thymic Density

Ordinal regressions were performed to examine the relationship of thymic scores with age, sex, and BMI. In thymus scoring, 25% NMOSD patients showed complete fatty replacement (score 0), and 50% NMOSD patients showed predominantly fatty thymus (score 1), whereas the proportion of HC group was 17% and 28%, respectively (*P* = 0.001, **Figure 4C**). Thymic scores showed an inverse correlation with age (95% CI, 1.075–1.325; *P* = 0.001) and BMI (95% CI, 1.123–1.964; *P* = 0.006). We observed no significant correlation of thymic scores with sex.

Next, we compared the thymic density between the two groups after disaggregating by age, sex, and BMI. Age (*P* < 0.0001; r = −0.637; **Figure 5E**) and BMI (95% CI, −4.109 to −1.271; *P* < 0.001; **Table 4**) showed a significant association with decline in thymus density. Thymic density in NMOSD patients was significantly lesser than that in the HC group (95% CI, 6.509–24.693; *P* = 0.001; **Table 4**). Moreover, partial correlation analysis after controlling for age and BMI revealed a marked association between thymic density and disease duration (*P* = 0.037; r = −0.278; **Figure 5F**), but no significant correlation between thymic predominant sides and disease duration (data not shown). There was no significant correlation of thymus density with sex.

The increased thymic fat and decreased thymic density in NMOSD patients indicate exacerbation of age-dependent thymus involution as compared with HC, which explains the age-related declining trend of thymic function.

There was no significant difference between anti-AQP4-positive and anti–AQP4-negative patients, with respect to thymic predominant sides and thymic density or thymic score.

Owing to the difficulty in distinguishing thymus fat from mediastinum, use of CT to measure thymus volume is not straightforward. Especially in patients with complete fatty involution, precise delineation of the thymic borders is mostly impossible. We are not sure whether the volume of the thymus has definitively decreased (data not shown).

### NMOSD Output Naive CD4 T Cells Decline With Age and Significantly Related to Thymic Involution and Predominant Sides Changes

**Figure 7:**
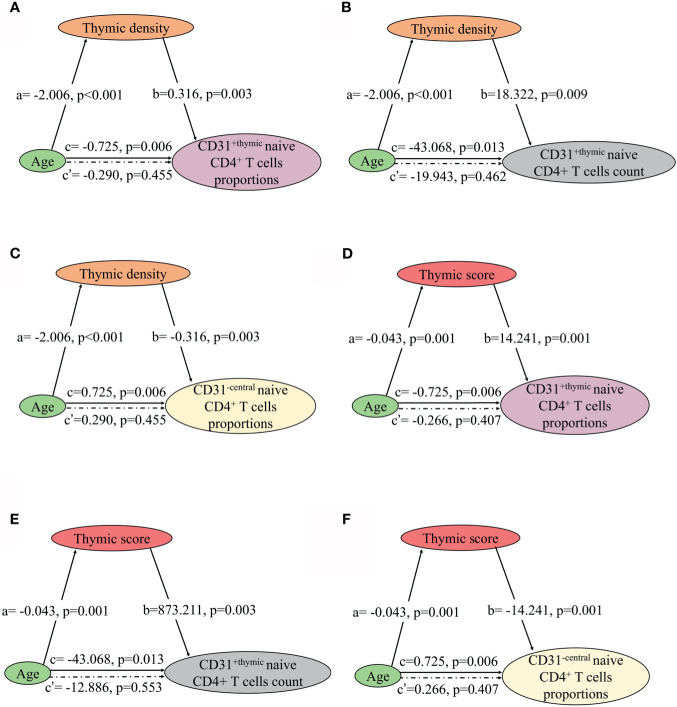
Mediation effects of changes in thymic density and score on the association of decline in NMOSD thymic output naive CD4 T cells with age. **(A–C)** Mediation analysis was performed to examine the potential indirect relationship between Age (X) and thymic output naive CD4 T cells (Y) *via* the thymic density (M). **(D–F)** Mediation analysis the potential indirect relationship between Age (X) and thymic output naive CD4 T cells (Y) *via* the thymic score (M). Path a and path b indicate the association between X and M, and M and Y, respectively. Path c represents the total effect of X on Y, and path c’ shows the direct effect of X on Y after controlling for mediating factor. X, predictor variable; Y, outcome variable; M, mediator.

Fourteen NMOSD patients underwent chest CT scan and flow cytometric analysis of naive CD4 T cells subpopulations simultaneously. We assessed the association of thymic density, score, and predominant sides with naïve CD4^+^ T- cell subpopulation in these patients. As expected, the proportions and count of CD31^+thymic^ naive CD4^+^ T cells increased with thymic density and score (**Figures 7A, B, D**, **E**), since increase in thymic fat and decreased thymic density indicate the trend of decline in thymic function. The decline in CD31^+thymic^ naive CD4^+^ T cells was associated with the decrease in thymic function, which can be regarded as a direct marker of thymic output (17). In contrast, the proportions of CD31^-central^ naive CD4^+^ T cells declined with thymic density and score (**Figures 7C**, **F**). There was no significant correlation of CD31^-central^ naive CD4^+^ T cell count with thymic density or score. As the count of CD31^-central^ naive CD4^+^ T cells remains stable over time, this implies a peripheral regulation independent of thymic activity (18). We observed no significant correlation of CD45RA^+^ CD62L^+^ naive CD4^+^ T cells with thymic density or score.

**Figure 8:**
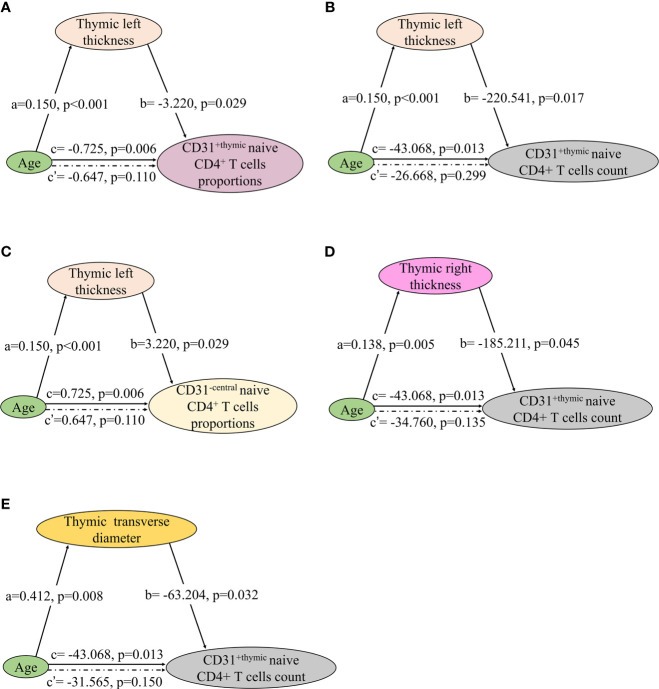
Mediation effects of changes in thymic predominant sides on the association of decline in NMOSD thymic output naive CD4 T cells with age. **(A–E)** Mediation analysis was performed to examine the potential indirect relationship between Age (X) and thymic output naive CD4 T cells (Y) *via* the thymic predominant sides (M). Path a and path b indicate the association between X and M, and M and Y, respectively. Path c represents the total effect of X on Y, and path c’ shows the direct effect of X on Y after controlling for mediating factor. X, predictor variable; Y, outcome variable; M, mediator.

The involution of thymus, characterized by disruption of thymic architecture and increase in adipocytes, contributes to the decrease in naïve T cell output. In thymic predominant sides, left thickness showed a significant association with the proportions and count of CD31^+ thymic^ naive CD4^+^ T cells and the proportions of CD31^−central^ naive CD4^+^ T cells (**Figures 8A–C**). Right thickness and transverse diameter showed a significant association with CD31^+thymic^ naive CD4^+^ T cell count (**Figures 8D**, **E**). There was no significant correlation of naive CD4^+^ T cells with thymic left length, right length, and anteroposterior diameter.

In the mediation analysis, both the thymic density and naive CD4^+^ T cells showed an inverse correlation age. In addition, the thymic density was found to fully mediate the relation between age and naïve CD4^+^ T cells (**Figures 7** and **8**).

## Discussion

To the best of our knowledge, this is the first study exploring the thymus characteristics and naive CD4 T-cell homeostasis in NMOSD. Thymus provides an inductive microenvironment for the differentiation, development, and maturation of T cells. In contrast, thymus involution, which is characterized by disruption of thymic architecture and increase in adipocytes, contributes to the decrease in naive T-cell output, which increases the risk of infection and autoimmune disease (9, 25–27). Increased thymic fat and decreased thymic density in patients with NMOSD indicated a trend of decline in thymic function. However, patients displayed a significantly higher fraction and absolute numbers of peripheral blood CD45RA^+^ CD62L^+^ naive CD4^+^ T cells.

Although it is clear that both the thymus and homeostatic proliferation contribute to naive T cell homeostasis and that their relative contributions change over time (7, 28), the functional consequences of the changes in naive T cell dynamics are not well characterized. The naive T-cell pool in mice was shown to be almost exclusively sustained by thymus output throughout the lifetime, whereas the adult human naive T-cell pool is almost exclusively maintained through peripheral T cell division (29, 30). Thymic involution has a minimal effect on the size of the peripheral T-cell pool (31), because of the extraordinary capacity of post thymic T cells to proliferate and maintain normal numbers of peripheral T cells. It is worth noting that higher IL-6 levels in peripheral blood can promote the differentiation of naive T cells into proinflammatory type 17 helper T cells. This may stimulate the compensatory proliferation of naive T cells, because patients with NMOSD simultaneously exhibit higher IL-6 levels and increased naive CD4^+^ T cells in the peripheral blood, and naïve T cells increase with IL-6 level significantly. The elevated homeostatic proliferation of naive T cells, as indicated by the increased absolute numbers of CD31^-central^ naïve CD4^+^ T cells, remains generally stable over time, implying a peripheral regulation independent of thymic activity.

In previous studies, patients with myasthenia gravis showed an association with NMOSD, or developed NMOSD after thymectomy (32). However, the association between thymus and pathogenesis of NMOSD is not well characterized. In this study, NMOSD patients showed exacerbated age-dependent thymus involution than HC, because the size, shape, and density of thymus showed significant changes in NMOSD patients. Anti-thymus AQP4-antibodies and AQP4 autoreactive T cells derived from thymus can trigger NMOSD, suggesting a strong linkage between thymus involution and the pathogenesis of NMOSD (33, 34). Thymus involution may initiate immunological response against AQP4 peptides, enhance the production of anti-AQP4 antibodies, aggravate the immune dysregulation, and impair self-tolerance; alternatively, it may be one of the main consequences of long-standing NMOSD.

Thymus plays a vital role in establishing T cell tolerance, which enables the initiation of immune responses to pathogens while avoiding autoimmune responses (35). The relative contributions of thymic and peripheral tolerance in the causation of CNS diseases are not well characterized. Studies of human CNS inflammation suggest a role for autoreactive T cells that have escaped thymic negative selection (36). In preclinical animal models, changes in thymic selection were associated with CNS autoimmunity (3). Thymic selection is underappreciated as a potential therapeutic target in CNS autoimmune disease and should be a focus of future research (37).

The differentiation of B cells into AQP4-IgG–producing plasmablasts along with IL-6 promotion is the primary pathogenetic mechanism of NMOSD (1, 12). Because IL-6 expression in human thymus increases with age and is associated with thymic involution (38), thymus degeneration maybe one of the causes of elevated IL-6 in NMOSD patients. Patients with NMOSD exhibit markedly elevated levels of IL-6 in the serum and cerebrospinal fluid (39–41). IL-6 receptor monoclonal antibodies satralizumab or tocilizumab were shown to significantly reduce the risk of NMOSD (1, 42, 43). The potential effect of IL-6 receptor monoclonal antibody on thymus degeneration provides a comprehensive understanding of the therapeutic mechanism.

There is no clear consensus as to whether the formation of adipocytes during thymic involution is a passive aggressive or active instigator of immunosenescence (44). Adipocytes in thymus can influence both the thymic and systemic immune function by secreting a diverse range of cytokines and hormones (45–47). Thus, further research is required to elucidate the role of thymic adipocytes in the establishment and maintenance of the T-cell repertoire. Approaches for thymic rejuvenation by preventing or slowing thymic adipogenesis may help regulate T-cell homeostasis in patients with NMOSD. Research shows that growth hormone administered in combination with metformin can reverse the immunosenescent trends by inducing regeneration of the thymus (48). As this potential novel strategy is feasible, we believe that reversing thymic involution to facilitate immune reconstitution is a viable therapeutic strategy for NMOSD, especially in patients with long-standing disease. Immune reconstitution to regulate T-cell homeostasis and IL-6 may also reduce the relapse of NMOSD.

Our results should be interpreted as preliminary because of some important study limitations, that is, lack of pathological data, cross-sectional study design, single-center scope, and small sample size of Asians. Large-scale multicenter studies with longitudinal observation and analysis of thymus autopsy data will provide more definitive evidence.

Overall, our study provides interesting insights that may further our understanding of the pathogenesis of NMOSD and indicate a potential novel strategy for patients with long-standing disease.

## Data Availability Statement

The original contributions presented in the study are included in the article/supplementary material. Further inquiries can be directed to the corresponding authors.

## Ethics Statement

The studies involving human participants were reviewed and approved by Beijing Tiantan Hospital, Capital Medical University. The patients/participants provided their written informed consent to participate in this study.

## Author Contributions

HaC, LY, and XZ conceptualized, designed experiments, interpreted results, and drafted the manuscript for intellectual content. HeC was involved in acquisition and analysis of the data. HW, LD, D-CT, YM provided administrative, technical, or material support. YX and YW performed the flow cytometric analysis. All authors contributed to the article and approved the submitted version.

## Funding

This work was supported by the Beijing Natural Science Foundation (No. 7162208), the National Key Research and Development Program of China (No. 2019YFC0121202) and Beijing Health and Technical Personal of High-level Plan (No. 2014-3-052).

## Conflict of Interest

The authors declare that the research was conducted in the absence of any commercial or financial relationships that could be construed as a potential conflict of interest.

## References

[1] Weinshenker BG Wingerchuk DM. Neuromyelitis Spectrum Disorders. MayoClinic Proc (2017) 92:663–79. 10.1016/j.mayocp.2016.12.014 28385199

[2] Zeka B Hastermann M Hochmeister S Kögl N Kaufmann N Schanda K. Highly Encephalitogenic Aquaporin 4-Specific T Cells and NMO-IgG Jointly Orchestrate Lesion Location and Tissue Damage in the CNS. Acta Neuropathologica (2015) 130:783–98. 10.1007/s00401-015-1501-5 PMC4654751 26530185

[3] Varrin-Doyer M Spencer CM Schulze-Topphoff U Nelson PA Stroud RM Cree BA. Aquaporin 4-Specific T Cells in Neuromyelitis Optica Exhibit a Th17 Bias and Recognize Clostridium ABC Transporter. Ann Neurol (2012) 72:53–64. 10.1002/ana.23651 22807325 PMC3405197

[4] Chaudhry M Velardi E Dudakov J van den Brink M. Thymus: The Next (Re)Generation. Immunol Rev (2016) 271:56–71. 10.1111/imr.12418 27088907 PMC4837659

[5] Chapman N Boothby M and Chi H. Metabolic Coordination of T Cell Quiescence and Activation. Nat Rev Immunol (2020) 20:55–70. 10.1038/s41577-019-0203-y 31406325

[6] Appay V Sauce D. Naive T Cells: The Crux of Cellular Immune Aging? Exp Gerontol (2014) 54:90–3. 10.1016/j.exger.2014.01.003 24440387

[7] Mold J Réu P Olin A Bernard S Michaëlsson J Rane S. Cell Generation Dynamics Underlying Naive T-Cell Homeostasis in Adult Humans. PloS Biol (2019) 17:e3000383. 10.1371/journal.pbio.3000383 31661488 PMC6818757

[8] Rosado-Sanchez I Herrero-Fernandez I Genebat M Ruiz-Mateos E Leal M Pacheco YM. Thymic Function Impacts the Peripheral CD4/CD8 Ratio of HIV-Infected Subjects. Clin Infect Dis (2017) 64:152–8. 10.1093/cid/ciw711 27986677

[9] Palmer S Albergante L Blackburn CC Newman TJ. Thymic Involution and Rising Disease Incidence With Age. Proc Natl Acad Sci USA (2018) 115:1883–8. 10.1073/pnas.1714478115 PMC5828591 29432166

[10] Lin J Li X and Xia J. Th17 Cells in Neuromyelitis Optica Spectrum Disorder: A Review. Int J Neurosci (2016) 126:1051–60. 10.3109/00207454.2016.1163550 26954363

[11] Kimura A Kishimoto T. IL-6: Regulator of Treg/Th17 Balance. Eur J Immunol (2010) 40:1830–5. 10.1002/eji.201040391 20583029

[12] Chihara N Aranami T Sato W Miyazaki Y Miyake S Okamoto T. Interleukin 6 Signaling Promotes Anti-Aquaporin 4 Autoantibody Production From Plasmablasts in Neuromyelitis Optica. Proc Natl Acad Sci USA (2011) 108:3701–6. 10.1073/pnas.1017385108 PMC3048150 21321193

[13] Kohler S Thiel A. Life After the Thymus: CD31+ and CD31- Human Naive CD4+ T-Cell Subsets. Blood (2009) 113:769–74. 10.1182/blood-2008-02-139154 18583570

[14] van den Broek T Borghans JAM Van Wijk F. The Full Spectrum of Human Naive T Cells. Nat Rev Immunol (2018) 18:363–73. 10.1038/s41577-018-0001-y 29520044

[15] De Rosa S Herzenberg L Herzenberg L Roederer M. 11-Color, 13-Parameter Flow Cytometry: Identification of Human Naive T Cells by Phenotype, Function, and T-Cell Receptor Diversity. Nat Med (2001) 7:245–8. 10.1038/84701 11175858

[16] Kohler S Wagner U Pierer M Kimmig S Oppmann B Möwes B. Post-Thymic *in vivo* Proliferation of Naive CD4+ T Cells Constrains the TCR Repertoire in Healthy Human Adults. Eur J Immunol (2005) 35:1987–94. 10.1002/eji.200526181 15909312

[17] Kohler S Keil T Alexander T Thiel A Swierzy M Ismail M. Altered Naive CD4(+) T Cell Homeostasis in Myasthenia Gravis and Thymoma Patients. J Neuroimmunol (2019) 327:10–4. 10.1016/j.jneuroim.2019.01.005 30686546

[18] Silva SL Albuquerque AS Matoso P Charmeteau-de-Muylder B Cheynier R Ligeiro D. IL-7-Induced Proliferation of Human Naive CD4 T-Cells Relies on Continued Thymic Activity. Front Immunol (2017) 8:20. 10.3389/fimmu.2017.00020 28154568 PMC5243809

[19] Yin C Pei XY Shen H Gao YN Sun XY Wang W. Thymic Homing of Activated CD4(+) T Cells Induces Degeneration of the Thymic Epithelium Through Excessive RANK Signaling. Sci Rep (2017) 7:2421. 10.1038/s41598-017-02653-9 28546567 PMC5445095

[20] Carvalho-Silva W Andrade-Santos J Souto F Coelho A Crovella S Guimarães R. Immunological Recovery Failure in cART-Treated HIV-Positive Patients Is Associated With Reduced Thymic Output and RTE CD4+ T cell Death by Pyroptosis. J Leukocyte Biol (2020) 107:85–94. 10.1002/jlb.4a0919-235r 31691351

[21] Araki T Sholl LM Gerbaudo VH Hatabu H Nishino M. Thymic Measurements in Pathologically Proven Normal Thymus and Thymic Hyperplasia: Intraobserver and Interobserver Variabilities. Acad Radiol (2014) 21:733–42. 10.1016/j.acra.2014.02.006 PMC4022310 24809315

[22] Araki T Sholl LM Gerbaudo VH Hatabu H Nishino M. Normal Thymus in Adults: Appearance on CT and Associations With Age, Sex, BMI and Smoking. Eur Radiol (2016) 26:15–24. 10.1007/s00330-015-3796-y 25925358 PMC4847950

[23] Shimamoto A Ashizawa K Kido Y Hayashi H Nagayasu T Kawakami A. CT and MRI Findings of Thymic Carcinoid. Br J Radiol (2017) 90:20150341. 10.1259/bjr.20150341 28106503 PMC5601511

[24] Simanovsky N Hiller N Loubashevsky N Rozovsky K. Normal CT Characteristics of the Thymus in Adults. Eur J Radiol (2012) 81:3581–6. 10.1016/j.ejrad.2011.12.015 22236705

[25] Takahama Y Ohigashi I Baik S Anderson G. Generation of Diversity in Thymic Epithelial Cells. Nat Rev Immunol (2017) 17:295–305. 10.1038/nri.2017.12 28317923

[26] Kadouri N Nevo S Goldfarb Y Abramson J. Thymic Epithelial Cell Heterogeneity: TEC by TEC. Nat Rev Immunol (2020) 20:239–53. 10.1038/s41577-019-0238-0 31804611

[27] James KD Jenkinson WE Anderson G. T-Cell Egress From the Thymus: Should I Stay or Should I Go? J Leukoc Biol (2018) 104:275–84. 10.1002/jlb.1mr1217-496r PMC6174998 29485734

[28] Okoye A Rohankhedkar M Konfe A Abana C Reyes M Clock J. Effect of IL-7 Therapy on Naive and Memory T Cell Homeostasis in Aged Rhesus Macaques. J Immunol (Baltimore Md: 1950) (2015) 195:4292–305. 10.4049/jimmunol.1500609 PMC4617185 26416281

[29] Johnson PL Yates AJ Goronzy JJ Antia R. Peripheral Selection Rather Than Thymic Involution Explains Sudden Contraction in Naive CD4 T-Cell Diversity With Age. Proc Natl Acad Sci USA (2012) 109:21432–7. 10.1073/pnas.1209283110 PMC3535632 23236163

[30] Thompson H Smithey M Uhrlaub J Jeftić I Jergović M White S. Lymph Nodes as Barriers to T-Cell Rejuvenation in Aging Mice and Nonhuman Primates. Aging Cell (2019) 18:e12865. 10.1111/acel.12865 30430748 PMC6351843

[31] den Braber I Mugwagwa T Vrisekoop N Westera L Mogling R de Boer AB. Maintenance of Peripheral Naive T Cells Is Sustained by Thymus Output in Mice But Not Humans. Immunity (2012) 36:288–97. 10.1016/j.immuni.2012.02.006 22365666

[32] Kimura K Okada Y Fujii C Komatsu K Takahashi R Matsumoto S. Clinical Characteristics of Autoimmune Disorders in the Central Nervous System Associated With Myasthenia Gravis. J Neurol (2019) 266:2743–51. 10.1007/s00415-019-09461-3 31342158

[33] Antoine JC Camdessanche JP Absi L Lassabliere F Feasson L. Devic Disease and Thymoma With Anti-Central Nervous System and Antithymus Antibodies. Neurology (2004) 62:978–80. 10.1212/01.wnl.0000115168.73299.88 15037705

[34] Chan KH Kwan JS Ho PW Ho SL Chui WH Chu AC. Aquaporin-4 Water Channel Expression by Thymoma of Patients With and Without Myasthenia Gravis. J Neuroimmunol (2010) 227:178–84. 10.1016/j.jneuroim.2010.07.016 20728226

[35] Yan F Mo X Liu J Ye S Zeng X Chen D. Thymic Function in the Regulation of T Cells, and Molecular Mechanisms Underlying the Modulation of Cytokines and Stress Signaling (Review). Mol Med Rep (2017) 16:7175–84. 10.3892/mmr.2017.7525 PMC5865843 28944829

[36] Held K Bhonsle-Deeng L Siewert K Sato W Beltrán E Schmidt S. αβ T-Cell Receptors From Multiple Sclerosis Brain Lesions Show MAIT Cell-Related Features. Neurol(R) Neuroimmunol Neuroinflamm (2015) 2:e107. 10.1212/nxi.0000000000000107 PMC4426681 25977934

[37] Handel AE Irani SR Hollander GA. The Role of Thymic Tolerance in CNS Autoimmune Disease. Nat Rev Neurol (2018) 14:723–34. 10.1038/s41582-018-0095-7 30451970

[38] Sempowski GD Hale LP Sundy JS Massey JM Koup RA Douek DC. Leukemia Inhibitory Factor, Oncostatin M, IL-6, and Stem Cell Factor mRNA Expression in Human Thymus Increases With Age and Is Associated With Thymic Atrophy. J Immunol (2000) 164:2180–7. 10.4049/jimmunol.164.4.2180 10657672

[39] Barros PO Cassano T Hygino J Ferreira TB Centurião N Kasahara TM. Prediction of Disease Severity in Neuromyelitis Optica by the Levels of Interleukin (IL)-6 Produced During Remission Phase. Clin Exp Immunol (2016) 183:480–9. 10.1111/cei.12733 PMC4750605 26472479

[40] Wei Y Chang H Li X Li H Li L Li H. Cytokines and Tissue Damage Biomarkers in First-Onset Neuromyelitis Optica Spectrum Disorders: Significance of Interleukin-6. Neuroimmunomodulation (2018) 25:215–24. 10.1159/000494976 30544111

[41] Uzawa A Mori M Masuda H Ohtani R Uchida T Sawai S. Interleukin-6 Analysis of 572 Consecutive CSF Samples From Neurological Disorders: A Special Focus on Neuromyelitis Optica. Clinica Chimica Acta; Int J Clin Chem (2017) 469:144–9. 10.1016/j.cca.2017.03.006 28283439

[42] Yamamura T Kleiter I Fujihara K Palace J Greenberg B Zakrzewska-Pniewska B. Trial of Satralizumab in Neuromyelitis Optica Spectrum Disorder. N Engl J Med (2019) 381:2114–24. 10.1056/NEJMoa1901747 31774956

[43] Zhang C Zhang M Qiu W Ma H Zhang X Zhu Z. Safety and Efficacy of Tocilizumab Versus Azathioprine in Highly Relapsing Neuromyelitis Optica Spectrum Disorder (TANGO): An Open-Label, Multicentre, Randomised, Phase 2 Trial. Lancet Neurol (2020) 19:391–401. 10.1016/s1474-4422(20)30070-3 32333897 PMC7935423

[44] Dixit VD. Thymic Fatness and Approaches to Enhance Thymopoietic Fitness in Aging. Curr Opin Immunol (2010) 22:521–8. 10.1016/j.coi.2010.06.010 PMC2993497 20650623

[45] Dixit VD. Impact of Immune-Metabolic Interactions on Age-Related Thymic Demise and T Cell Senescence. Semin Immunol (2012) 24:321–30. 10.1016/j.smim.2012.04.002 22546243

[46] Procaccini C Pucino V Mantzoros C Matarese G. Leptin in Autoimmune Diseases. Metabolism: Clin Exp (2015) 64:92–104. 10.1016/j.metabol.2014.10.014 25467840

[47] Tan J Wang Y Wang S Wang S Yuan Z Zhu X. Label-Free Quantitative Proteomics Identifies Transforming Growth Factor β1 (TGF-β1) as an Inhibitor of Adipogenic Transformation in OP9-DL1 Cells and Primary Thymic Stromal Cells. Cell Bioscience (2019) 9:48. 10.1186/s13578-019-0311-1 31249661 PMC6570845

[48] Fahy GM Brooke RT Watson JP Good Z Vasanawala SS Maecker H. Reversal of Epigenetic Aging and Immunosenescent Trends in Humans. Aging Cell (2019) 18:e13028. 10.1111/acel.13028 31496122 PMC6826138

